# Re-evaluating the *ordre public* bar to cannabis patents in South Africa after decriminalisation

**DOI:** 10.1186/s42238-026-00488-8

**Published:** 2026-08-14

**Authors:** Lonias Ndlovu

**Affiliations:** School of Law, University of Venda, Private Bag X5050, Thohoyandou, Limpopo, 0950 South Africa

**Keywords:** Cannabis patentability in South Africa, Section 25(4)(a) of the Patents Act, Ordre public and morality in patent law, Cannabis genetic resources, WIPO GRATK Treaty, ARIPO patent system, Substantive search and examination

## Abstract

**Background:**

South African law treats different activities involving cannabis in distinct ways. *Prince* protects an adult's private use, possession, and cultivation for personal consumption, but the law still prohibits dealing and other activities. Parliament enacted the Cannabis for Private Purposes Act 7 of 2024, which currently awaits a presidential proclamation to take effect. This review assesses whether this legal framework causes every cannabis-related invention to fall under s 25(4)(a) of the Patents Act 57 of 1978.

**Main body:**

This review doctrinally analyses s 25(4)(a) of South Africa's Patents Act. It compares that provision with the Agreement on Trade-Related Aspects of Intellectual Property Rights (TRIPS), the European Patent Convention (EPC), and materials from the African Regional Intellectual Property Organization (ARIPO). It also uses selected public patent records to test what available databases disclose. The analysis separates the moral controversy over cannabis, the legality of particular conduct, and the statutory effect of publishing or exploiting the claimed invention. The *Prince* decision addresses only part of this legal context. However, its privacy holding makes blanket criminality an unviable basis for refusing all cannabis patents. Section 25(4)(a) directs the inquiry into whether publishing or exploiting a particular invention would generally be expected to encourage offensive or immoral behaviour. The review also considers agrarian participation, genetic resource disclosure, and South Africa's unfinished move from depository registration to substantive search and examination.

**Conclusion:**

Partial decriminalisation does not automatically entitle a cannabis invention to patent protection. Conversely, moral debate or strict regulation surrounding cannabis does not justify an automatic refusal. Standard patentability requirements perform most of the screening work. However, s 25(4)(a) remains available if publishing or exploiting a specific invention would generally encourage offensive or immoral conduct. Patent administration requires simultaneous improvement through rigorous examination, clear public records, and dependable safeguards for biodiversity, origin disclosure, and benefit sharing.

## Background

Cannabis has moved beyond the scope of criminal law. The plant serves as an agricultural crop, a controlled substance, and a topic of pharmaceutical and technical research. Commercial forecasts vary and often lean toward optimism, but they demonstrate sustained economic interest (Grand View Research 2030). Public health reports remain cautious, classifying cannabis as the world's most widely used controlled drug (United Nations Office on Drugs and Crime 2025). South African policy seeks to develop a cannabis and hemp value chain, while Lesotho actively promotes medicinal cannabis to attract investment (Western Cape Department of Agriculture 2023; Government of South Africa 2025; Lesotho National Development Corporation 2025). European regulatory sources document continuous consumption alongside statutory reforms (European Union Drugs Agency 2024). Patent law plays an active role in this expanding market.

Patent law affects how value is distributed. The key questions are who controls useful technology, where processing and research occur, and whether growers and communities remain confined to supplying raw material. Research on small-scale farmers in South Africa and literature on agrarian reform in Lesotho and Zimbabwe show that licensing can concentrate capital, regulatory access, and downstream knowledge elsewhere (Dube I et al. 2023; Bloomer 2019; Thetsane 2024; Rusenga et al. 2024). Wyse and Luria find that much cannabis IP activity relates to commercially useful technologies and plant-related rights (Wyse and Luria 2021). In my view, entering a formal market does not by itself protect indigenous growers.

Patents promote research and public disclosure, but claims based on local genetic material or traditional knowledge can place control of related inventions in foreign hands. South African biodiversity and benefit-sharing law addresses part of this risk. Substantive patent examination and transparent public records can address another part. Section 25 (4)(a) cannot perform all three functions.

The legal starting point is s 25(4)(a) of the Patents Act, not the general reputation of cannabis. A long history of prohibition may make a cannabis invention seem morally tainted, but this impression cannot be the statutory test. In *Minister of Justice and Constitutional Development v Prince*, the Constitutional Court held that prohibiting an adult’s private use, possession for personal consumption, and private cultivation for that purpose unjustifiably limits privacy rights (paras 1–2, 99–101 and 109) (Minister of Justice and Constitutional Development and Others v Prince 2018).

*Prince* did not declare cannabis moral, nor did it open a general commercial market. It protected a specific area of adult private life and conduct. Parliament later enacted the Cannabis for Private Purposes Act 7 of 2024. However, s 8 of the Act leaves its commencement to a future presidential proclamation (Cannabis for Private Purposes 2024). No such proclamation had appeared in official materials by 13 August 2026. The Department of Justice had instead published draft regulations for comment in February 2026 (Department of Justice and Constitutional Development 2026). Therefore, the legal protection for private use still rests on the *Prince* decision.

The earlier *Prince* litigation sought a religious exemption. Lubaale and Mavundla explain the later shift to a privacy argument, which resulted in the 2018 judgment (Lubaale and Mavundla 2019). This shift did not settle the morality of cannabis, but it altered the legal basis for the patent debate. Once South African law began to protect some cannabis conduct while prohibiting other conduct, criminality could no longer justify the refusal of every cannabis-related invention by the Companies and Intellectual Property Commission (CIPC).

The resulting patent issue is specific and narrow. Privacy, criminal prohibition, and patentability answer different questions. Under s 25(4)(a), the inquiry is about the expected effect of publishing or exploiting the invention claimed. This effect may involve criminal conduct.

This review asks whether a blanket exclusion for all cannabis inventions can survive this distinction and, if not, what safeguards are needed. I argue that it cannot. This argument does not depend on finding that cannabis is morally acceptable. Instead, it flows from s 25(4)(a), the distinction between a patent and regulatory permission, and the need to examine each patent application on its own merits.

Such a patent dispute is also interdisciplinary. Patent governance can affect whether value from research and processing remains in South Africa. It can also determine whether applicants disclose genetic resources and whether small producers can identify the patent rights they face. Therefore, patent doctrine operates within the wider cannabis economy. This review connects the morality question to comparative regulation, ARIPO practice, public patent records, indigenous knowledge, and South Africa's planned introduction of substantive search and examination.

## Main text

### Search method and sources

This is a narrative and critical legal review. It combines doctrinal analysis, functional comparison, and a limited search of public patent sources. It is not a systematic review or a meta-analysis. Therefore, PRISMA reporting is not applicable.

The author used ChatGPT during preparation and revision to generate preliminary ideas and locate sources, Claude to redraft and edit language, and Codex to check journal style and audit citations. The author treated none of these tools as an independent legal authority. The author verified all legal authorities and patent records, refined the arguments, and takes full responsibility for the contents of this review.

The doctrinal analysis rests on s 25 of the Patents Act, *Prince*, the Cannabis for Private Purposes Act, art 27 of the TRIPS Agreement, and arts 53(a) and 53(b) of the EPC. It also considers the 2024 World Intellectual Property Organization (WIPO) Treaty on Intellectual Property, Genetic Resources and Associated Traditional Knowledge (GRATK Treaty). Non-patent constitutional cases serve only as analogies supporting specific, limited arguments.

The comparison is functional rather than encyclopaedic. The European Patent Office (EPO) is included because it has a well-developed body of case law on morality, *ordre public*, and plant-related exclusions. Lesotho, Zimbabwe, Malawi, and Ghana were chosen because each country illustrates a different problem that South Africa also faces. These problems include export-oriented licensing, agrarian inclusion, disclosure of genetic resources, and controlled industrial regulation. All four countries are parties to the ARIPO Harare Protocol, which makes the relationship between regional and national law important.

Sections 46 and 48 of the 2025 Harare Protocol divide responsibility between ARIPO and a designated state. ARIPO conducts the substantive patent examination and may grant the regional patent. Before the grant, a designated state can either communicate its acceptance under s 46(2)(a) or state under s 46(2)(b) that the patent will have no effect in its territory. If the state does not object, s 48(2) gives the grant effect in that state (African Regional Intellectual Property Organization 2025, 2026). Consequently, domestic law remains relevant for opposition and enforcement, even though the Protocol does not always require a separate national confirmation.

Article 2(2) of the EPC achieves a similar result through a different route. In each designated contracting state, a European patent has the same effect as a national patent and is subject to the same conditions (European Patent Office 2024, 2026). Although the ARIPO and EPC systems are not interchangeable, both show that regional examination operates through domestic law, not outside it.

I used the patent records for a deliberately limited task. The search identifies selected publications, notes the legal statuses visible in the databases, and allows another reader to repeat the search on the stated dates. It cannot measure national portfolios, ownership, patent quality, licensing, or commercial success. None of the doctrinal conclusions depends on the number of patents retrieved.

Google Patents provided the bibliographic and family information for selected examples, while WIPO PATENTSCOPE enabled verification of international publications (Google. 2026; World Intellectual Property Organization. 2026). I cross-checked these results against ARIPO publications and CIPC records where possible (African Regional Intellectual Property Organization 2024; Companies and Intellectual Property Commission 2025, 2026). These public databases help trace specific filings, but they do not constitute an official national census.

The primary search identified publications from 1 January 2018 to 31 December 2025. I retained one older South African filing to determine whether cannabinoid publications preceded *Prince*. Initial searches took place from 8 to 12 April 2026, with checks on 14 May 2026 and a final audit on 22 July 2026. The search query was: (cannabis OR cannabinoid OR cannabinoids OR hemp OR THC OR CBD OR marijuana OR Cannabis sativa). Combining these terms with ZA, LS, ZW, MW, GH, and AP/ARIPO filters isolated relevant African records. EPC and Patent Cooperation Treaty (PCT) filters retrieved non-African examples.

A record was included if the title, abstract, or indexed family information showed an express link between the claimed subject matter and cannabis, cannabinoids, hemp, cannabis genetics, or a plant trait. I excluded incidental references and records that lacked enough bibliographic information to identify the publication or family. Where family data existed, I deduplicated by patent family. Otherwise, I used the publication number for the jurisdiction. However, the sample remains illustrative.

The databases have real limitations. National data feeds differ in timing and coverage, and title and abstract searches can miss material disclosed only in the claims or specification. Family and status fields may be incomplete or delayed. Hence, an aggregator’s label is not a legal opinion. Therefore, I draw no conclusions about present ownership, licensing, prosecution history, enforceability, prevalence, growth, or comparative performance. A record that does not appear in this search was simply not found by this particular search method.

### The statutory problem: Sect. 25 of the Patents Act

Section 25(1) states the basic rule that a patent may be granted for a new invention that involves an inventive step and can be used or applied in trade, industry, or agriculture (Act and 57 of 1978). Novelty and the state of the art are covered in ss 25(5)-(8), while s 25(10) addresses inventive step. Sections 25(11) and (12) concern methods of treatment or diagnosis and the related product exception. These latter provisions are not general rules on novelty, inventive step, or industrial applicability.

Section 25(4)(a) bars the grant of a patent for an invention where its publication or exploitation would generally encourage offensive or immoral behaviour (Act and 57 of 1978). The Act does not use the widely recognised concept of *ordre public*. That concept appears in art 27(2) of TRIPS and art 53(a) of the EPC. This review uses the term solely as a comparative framework. It must not be confused with the explicit South African statutory text (Convention on the Grant of European Patents 2026).

Three legal questions arise and must be kept separate. First, cannabis may attract moral controversy. Second, particular cannabis conduct may be prohibited, permitted, or constitutionally protected. Third, publishing or exploiting the claimed invention may generally be expected to encourage the behaviour mentioned in s 25(4)(a). Only the third question is the statutory patent question. Neither moral controversy nor regulatory illegality can answer it without further analysis.

Section 25(4)(a) requires an inquiry into effects. It does not exclude every invention associated with a controversial subject, nor does it ask only whether the plant or practice attracts moral disapproval. The required link is between publishing or exploiting the particular invention and the behaviour described in the section. A mere association with cannabis is too remote.

Even if one assumes, for the sake of argument, that cannabis is inherently immoral, publishing a claim to an analytical method, pharmaceutical composition, or technical process would not automatically encourage immoral behaviour. Section 25(4)(a) still requires the expected effect of encouraging immoral or offensive behaviour to be established. Social utility is not an independent ground for getting a patent, although lawful technical uses may rebut an inference that the invention encourages such behaviour.

Section 14 of the Constitution guarantees the right to privacy (Constitution of the Republic of South Africa 1996). *Barkhuizen v Napier* is instructive. The Court located public policy in constitutional norms, not in free-standing moral intuition (paras 28–30) (Barkhuizen v Napier 2007). Although the case concerns contract law, it confirms that a South African decision maker who invokes public policy cannot ignore the Constitution.

The Constitutional Court addressed trade marks and free speech in *Laugh It Off Promotions CC v South African Breweries International*. It refused to infer harm without a clear factual basis (paras 54–57) (Laugh It Off Promotions CC v South African Breweries International (Finance) 2006). This test cannot be transplanted directly into s 25(4)(a). The judgment nevertheless shows that moral disapproval cannot replace the factual connection required by the governing legal rule.

*Affordable Medicines Trust v Minister of Health* is also relevant, but to a more limited extent. The Court recognised that regulating medicines serves safety and access goals and insisted that public power must stay within the limits of the empowering statute (paras 21–23 and 49–50) (Affordable Medicines 2006). The case is not a patent law authority. However, it reminds us that specialised regulations separate from patent law govern therapeutic products.

Workplace decisions illustrate the same need for context. In *Enever v Barloworld Equipment South Africa*, the Labour Appeal Court considered a positive cannabis test without evidence of workplace impairment. It held that the zero-tolerance policy unfairly discriminated against the employee (paras 38, 52 and 54) (Enever v Barloworld Equipment South Africa, A Division of Barloworld South Africa (Pty) Ltd (JA86, 22) 2024). A case note by Mbiada et al., which criticises the judgment, separately examines use, testing, impairment, and dependency (Mbiada et al. 2024). These sources confirm that specific evidence about the conduct determines the legal consequence, rather than the generic label cannabis.

Patentability must also be separated from permission to use an invention (licence). Section 45(1) gives the patentee exclusionary rights, including the right to 'exclude other persons from making, using, exercising, disposing or offering to dispose of, or importing the invention' (Act and 57 of 1978). A patent grant does not authorise cultivation, manufacture, sale, prescription, possession, or use in breach of other laws. Regulatory authorities may prohibit conduct described in a patent. Conversely, lawful conduct does not guarantee a valid patent. This distinction substantially weakens any attempt to turn criminal or regulatory rules into a categorical patent bar.

The patent office must rely on novelty, inventive step, and statutory exclusions to eliminate weak claims. Lack of novelty destroys anticipated subject matter, whereas s 25(10) invalidates obvious variations. Specific statutory rules govern plant varieties, treatment methods, and unsupported claims. Decision makers must apply these dedicated provisions rather than using morality as a shortcut around proper searching or analysis of claim scope and sufficiency.

Section 25(4)(a) serves its own purpose. On proper evidence, a decision maker may conclude that publishing or exploiting the claimed invention would generally be expected to encourage offensive or immoral behaviour. That conclusion must stem from the specific invention and its effect under the statute. It cannot be based on abstract concerns about cannabis.

### Privacy-based decriminalisation and the limits of categorical patent exclusion

The *Prince* decision altered the constitutional baseline on privacy grounds (Minister of Justice and Constitutional Development and Others v Prince 2018). It protects an adult’s private use or possession for personal consumption and private cultivation for that purpose. The court did not rule on commercial dealing, an unrestricted industry, or the moral status of cannabis. Therefore, it is correct to argue that constitutionally protected conduct can remain morally contested.

This observation leaves the patent issue unresolved. Criminal prohibition offered an unreliable proxy for the standards in s 25(4)(a) long before the *Prince* decision. Current constitutional protections for certain cannabis activities render that proxy even less defensible. Decriminalisation reshapes the legal framework, but moral disagreement still persists.

South African law currently protects some private conduct but continues to prohibit dealing and other activities. Parliament enacted the 2024 Act, but it had not commenced at the time of writing (Minister of Justice and Constitutional Development and Others v Prince 2018; Cannabis for Private Purposes 2024; Department of Justice and Constitutional Development 2026). This mixed framework provides no single moral answer for the patent office. Section 25(4)(a) must supply the legal test.

The patent office must therefore apply standard technical scrutiny to cannabis applications. A claim for a composition, analytical method, delivery device, genetic marker, or process may fail under rules on novelty, inventive step, sufficiency, excluded subject matter, or s 25(4)(a). Examiners cannot refuse an application simply because the specification mentions cannabis.

A morality review will sometimes be relevant. The facts may establish that publishing or exploiting the invention would generally be expected to encourage the behaviour named in the Patents Act. Inventing dramatic examples in advance serves no useful purpose. The decision maker should identify the claimed invention, the expected way it would be published or exploited, the behaviour said to follow, and the evidence for that conclusion. This is a demanding exercise, but it is no more demanding than the section itself requires.

### TRIPS, the EPO and the proper scope of morality review

#### TRIPS

Art 27(1) of TRIPS mandates patent protection across all fields of technology, provided the invention meets standard criteria for novelty, inventive step, and industrial applicability. Art 27(2) allows members to exclude subject matter when preventing commercial exploitation protects *ordre public*, morality, human, animal, or plant life, or the environment (Agreement on Trade-Related Aspects of Intellectual Property Rights 1994). The provision explicitly prevents members from excluding an invention solely because domestic law forbids its exploitation.

Art 27(2) of TRIPS and s 25(4)(a) of South Africa’s Patents Act ask different questions. The former addresses the necessity of preventing commercial exploitation for a stated public interest. The latter deals with the expected encouragement of offensive or immoral behaviour through publication or exploitation. One restraint is common to both, since illegality alone is insufficient. The *Prince* decision reinforces that point factually but did not create it.

### The EPO comparison: plant exclusions, evidence and lawful exploitation

Decisions and Guidelines from the European Patent Office (EPO) do not bind the CIPC or a South African court. Their comparative value is in showing how a mature examining system identifies the proper statutory objection instead of letting morality absorb questions of plant variety and technical patentability.

G 1/98 concerned art 53(b) and plant varieties, not morality (G 0001/98 (Transgenic plant/NOVARTIS II) 2026; World Intellectual Property Organization 2026). The Enlarged Board of Appeal accepted that a claim which could embrace plant varieties is not excluded if it is not directed to an individually identified variety as such. This reasoning matters in the cannabis field. A claim to a genetic marker, transformed plant, laboratory process, or extraction technique may raise a technical or plant subject matter question without being a claim to a variety. South African law should identify that question directly.

The decision in T 315/03 directly applied art 53(a) (T 0315/03 (Transgenic animals/HARVARD) 2026). The Board required evidential support for the asserted moral objection and assessed morality at the filing or priority date. South Africa can draw a limited comparative lesson from this framework without adopting European burdens of proof. Assertions of offensiveness cannot replace the analysis required by the South African provision.

The 2026 EPO Guidelines confirm that art 53(a) allows patents for inventions that permit non-offending uses (European Patent Office 2026). Researchers, medical professionals, testing facilities, and industrial hemp producers utilise cannabis technologies even while domestic law bans other uses. An illicit application does not characterize the entire invention. This comparative analysis offers guidance, but s 25(4)(a) remains the binding statutory authority.

This comparative analysis urges judicial restraint. Both TRIPS and the EPO framework confine morality arguments strictly to their enabling provisions. Political controversy, potential misuse, or separate statutory controls do not expand these limited legal exceptions.

### Comparative African trajectories and institutional nuance

Lesotho, Zimbabwe, Malawi, and Ghana take distinct approaches to combining ARIPO patent grants with domestic cannabis regulation. Examining these models reveals how commercial licensing, local exclusions, and regional patents interact to affect domestic technology control. Available records support a qualitative comparison of these frameworks.

### ARIPO does not erase national law

Ghana, Malawi, Lesotho, and Zimbabwe belong to the Harare Protocol states. Section 6 of the 2023 text originally contained the no-effect mechanism (Lesotho 2023), which now sits in ss 46 and 48 of the 2025 consolidated text (African Regional Intellectual Property Organization 2025, 2026). ARIPO examines and grants the application. A designated state can then notify ARIPO that the patent lacks effect within its borders. Lacking such notification, the regional grant automatically operates as a national patent enforced through domestic courts. ARIPO acts as more than a simple filing system, and its regional examination leaves national law fully operational.

A cannabis application can therefore be assessed at both regional and national levels. A designated state can communicate that a regional grant will have no effect under its own law. Silence has a different consequence because the grant takes effect without an additional national validation step. Writers misstate the Protocol when they treat either the regional or national framework as self-contained.

### Lesotho

Lesotho licensed medicinal cannabis early and continues to recruit foreign investors for the industry (Lesotho National Development Corporation 2025). Section 5(8) of the Industrial Property Order 1989 uses broad terms, excluding inventions that violate public order or morality from patentability (Lesotho 1989). This text differs from South Africa's expected encouragement standard. Authorities evaluating an ARIPO application designating Lesotho must assess it under both legal instruments. Sectoral research highlights high entry barriers, heavy reliance on external capital, and weak local ownership (Bloomer 2019; Thetsane 2024). A cultivation licence fails to prove domestic patent ownership. South Africa must not mistake expanding commercial cultivation for growing domestic technological capacity.

### Zimbabwe

Zimbabwe permits commercial cannabis production for scientific and medicinal uses through the Medicines Control Authority (Medicines Control Authority of Zimbabwe 2026). Its patent statute offers the closest regional match to South African law. Section 13(1)(b1) authorises refusal when an invention's use would generally endanger public order or encourage offensive, immoral, or anti-social conduct. Section 13(2) prohibits refusal simply because commercial use violates Zimbabwean law (Zimbabwe 2026). The statute separates illegal conduct from the expected effects of an invention's use. This distinction independently supports the interpretation of South Africa's s 25(4)(a) in this review. Analysts must separate this legal principle from industry distribution patterns. Current research highlights continuing land inequality and limited local participation (Rusenga et al. 2024). The public sources reviewed here do not establish who owns the relevant processing technologies.

### Malawi

Malawi’s Cannabis Regulation Act 2020 regulates medicinal cannabis and industrial hemp (Malawi 2020). Under the older Patents Act, the Registrar may refuse an application where use of the invention would be contrary to law or morality (Malawi 2026). The provision is directed to use, although it lacks South Africa’s expected encouragement language. Malawi also became the first state to ratify the GRATK Treaty (World Intellectual Property Organization 2024). No domestic cannabis patent portfolio can be inferred from those facts. They instead show why patent examination and origin disclosure should accompany commercialisation, especially where research draws on landraces or community knowledge.

### Ghana

Ghana restricts cannabis use to medical and industrial sectors, sets a 0.3 per cent THC ceiling, and mandates licensing by the Narcotics Control Commission (Narcotics Control Commission (Ghana) 2026). The Patents Act 2003, as ammended by the Patents Ammendent Act 2020, incorporates TRIPS principles, as s 3(2)(d) bars patents only when stopping commercial exploitation protects public order or morality (Ghana 2003). Strict market regulation works alongside a specific patent assessment. Ghana matters to this study because it maintains that precise legal separation.

### What the comparison shows

This comparison demonstrates that member states lack a unified morality standard. Lesotho applies broad exclusions, Zimbabwe separates statutory illegality from expected practical effects, Malawi retains traditional law-and-morality language, and Ghana uses the TRIPS necessity test. Industrial structures vary widely across these nations. Regulatory licensing fails to demonstrate local technological ownership. ARIPO examination leaves domestic exclusions fully intact. Regulated markets can increase disputes over plant origin and benefit sharing. South Africa must therefore avoid blanket patent bans while refusing to assume that patent growth automatically delivers inclusive development.

### The EPC interface with national law

Article 2(2) EPC provides a helpful system comparison. A granted European patent functions in each designated state as a national right controlled by domestic law (European Patent Office 2024, 2026). Regional examiners apply art 53(a) during examination, but national territorial rules take over after grant. ARIPO faces similar structural challenges, although its specific rules and member state procedures differ from the EPC model.

### Genetic resources, indigenous strains and the WIPO GRATK Treaty

South Africa signed the WIPO GRATK Treaty of 2024 but has not ratified or acceded to it (World Intellectual Property Organization 2024). WIPO records from 13 March 2026 showed Albania, Malawi, and Uganda as the three states depositing instruments, leaving the treaty inactive (World Intellectual Property Organization 2026). The treaty serves as an evolving standard for South Africa rather than a binding patent commitment.

Art 3 will require a contracting party, once bound, to demand disclosure when a claimed invention is based on genetic resources or associated traditional knowledge. Depending on what is known, the applicant must identify the country of origin or source of the genetic resource, or the Indigenous Peoples or local community providing the associated knowledge (World Intellectual Property Organization 2024). An applicant who knows none of this information must make a declaration to that effect.

That requirement is directly relevant to South African cannabis research. The literature records long-standing local cultivation and knowledge associated with South African dagga (Oriakhogba et al. 2024). Patent administration should therefore consider origin information when a claimed invention relies on a local landrace, genetic resource, or associated knowledge.

Observers must not overstate the impact of the Treaty. Art 5.3 stops a contracting party from revoking or invalidating a patent solely for missing disclosure information, subject to the fraud exception in art 5.4. However, art 5 requires effective measures for non-compliance and allows stronger penalties when national law shows fraudulent conduct or intent (World Intellectual Property Organization 2024).

South Africa already has a domestic framework. The National Environmental Management: Biodiversity Act and the 2015 Bioprospecting, Access and Benefit-Sharing Amendments Regulations govern permits, material transfer, and benefit sharing in relevant cases (Environmental and Management: Biodiversity Act 10 of 2004; Amendments to the Regulations on Bioprospecting, Access and Benefit-Sharing, General Notice 447 of 2015). Patent validity is only one possible issue. A dispute may also concern lawful access, the identity of the provider, contractual benefit sharing, or compliance with permit requirements.

South Africa can address biodiversity compliance outside the morality enquiry. If South Africa implements the Treaty, CIPC will need a workable system to record the required origin or source information. The Treaty does not oblige patent offices to verify the authenticity of that disclosure. Domestic permit, prior consent, material transfer, and benefit-sharing duties remain independently enforceable where they apply.

A total patent ban may seem to protect indigenous rights, but it may also obscure the more direct legal questions. Applicants may seek patents abroad, leaving no corresponding entry in South Africa's register. Origin disclosure, biodiversity permits, and benefit-sharing rules provide more targeted safeguards than a categorical morality exclusion.

## Public visibility of cannabis patent records: an illustrative exercise

### Purpose and caution

Patent counts could suggest a precision that these searches cannot support. This review uses the records to identify published filings, examine specific claims, and check details against named sources. The data prove neither a national trend nor a filing gap between Africa and Europe.

### South African records located

The search results yielded three South African B-kind publications. A B-kind publication identifies a patent document associated with a South African grant, but the public database lacked reliable data on current enforceability. Each record is therefore treated as a published grant document and family member, not as proof of a presently enforceable local right.

ZA201906516B, *Process for purification and separation of cannabinoids from dried hemp and cannabis leaves*, was published on 26 August 2020. It is the South African member of a family that includes WO2019010419A1. The indexed South African status was recorded as unknown during re-auditing, so no inference is made about its current enforceability (ZA201906516B 2026).

ZA202205648B, published on 25 January 2023, bears the title Cannabinoid nanomicelle preparation and method for preparing same. The same patent family contains WO2021109823A1. The indexed South African record lacked clear status data, so this review draws no conclusions on its present legal force (ZA202205648B 2026).

The older record, ZA200802767B, *Process for production of delta-9-tetrahydrocannabinol*, was published on 30 September 2009 and belongs to the family containing WO2007041167A2. Its inclusion shows that cannabinoid-specific publication preceded *Prince*. The public record does not establish whether the South African right is presently enforceable (ZA200802767B 2026).

These three documents confirm publication, not patent quality or domestic ownership. They do not establish whether the claims were valid when granted, whether renewal fees were paid, or whether the rights are now enforceable. Those questions require the relevant official file and evidence from the register.

### EPC and PCT examples

The non-South African records outlined next are only examples. EP1536810B1, *Extraction of pharmaceutically active cannabinoids from plant materials*, was granted and published on 1 August 2012. Its family includes WO2004016277A2, and the indexed European status is expired after its full term (EP1536810B1 2026). EP2161262A1, *THCV and THCV extracts derived from plant material*, is an application publication dated 10 March 2010, and its indexed status is withdrawn (EP2161262A1 2026). The PCT record WO2024227244A1, *Genetic markers for powdery mildew resistance in cannabis and related methods*, was published on 7 November 2024. It is not a granted patent, and the indexed PCT application is recorded as ceased (WO2024227244A1 2026).

Wyse and Luria cover a far broader range of downstream technologies and plant variety rights (Wyse and Luria 2021). This review does not attempt to reproduce that global overview. Instead, the analysis focuses on the specific records that these targeted searches uncovered within the chosen jurisdictions.

The searches yielded no reliable national patent sets for Lesotho, Zimbabwe, Malawi, or Ghana to support portfolio claims. This could be due to database coverage, indexing, filing routes, the search design, or genuinely limited activity. The evidence cannot distinguish among these reasons. Therefore, absence from these results does not prove absent filings or weak innovation. Table 1 summarises these limited findings.

**Table 1:** Public-source visibility of selected cannabis-related patent records

Setting	Patent/regulatory setting	What the defined search located	Permissible inference
South Africa	National depository system; SSE implementation in progress	ZA201906516B, ZA202205648B and ZA200802767B; publication and family details audited in the text	Publication is confirmed. No conclusion can be drawn about total filings, validity, ownership, current enforceability, or trends
Lesotho	National law and the ARIPO route apply. The medicinal sector is licensed	The search did not find a reliable national set of cannabis-specific patents	No conclusion can be drawn about the absence of filings, innovation, or domestic ownership
Zimbabwe	National law and the ARIPO route apply. The medicinal and scientific sector is licensed	The search did not find a reliable national set of cannabis-specific patents	No conclusion can be drawn about the absence of filings, innovation, or domestic ownership
Malawi	National law and the ARIPO route apply. Medicinal and industrial cannabis are regulated, and Malawi has ratified the GRATK Treaty	The search did not find a reliable national set of cannabis-specific patents	Treaty leadership does not confirm a national cannabis patent portfolio
Ghana	National law and the ARIPO route apply. There is a controlled medical and industrial regime	The search did not find a reliable national set of cannabis-specific patents	Licensing and THC controls do not confirm patent volume or ownership
EPC/PCT examples	Substantive examination at the EPO. International PCT publication	EP1536810B1 (expired grant), EP2161262A1 (withdrawn application) and WO2024227244A1 (PCT publication, ceased application)	This is for qualitative illustration only. The records have different routes and legal statuses

Table 1 shows what the search found and what it did not find. The table is not a ranking. It does not measure filings, ownership, claim quality, domestic value, or commercial success. The practical point is simple. Researchers and affected communities need patent information that they can search, find, and understand legally.

### Weak patents, depository grant and the transition to substantive search and examination

A narrow morality exclusion puts more weight on patent quality. South Africa is still largely a depository system, where a patent can be granted for formal compliance without a full prior art search or a substantive review of novelty and inventive step (Companies and Intellectual Property Commission 2026). New investment in cannabis makes the cost of broad or weak claims more immediate.

This weakness is not unique to cannabis. In any fast-moving field, a non-examining office might register claims that are anticipated, obvious, insufficiently supported, or directed to excluded subject matter. Cannabis applications, for example, range from formulations and analytical tools to processing methods, genetics, and plant traits. If validity is tested only during litigation, distinguishing a genuine invention from a weak claim becomes much more expensive.

CIPC’s own documents describe substantive search and examination (SSE) as a work in progress. The revised Strategic Plan 2025/26–2029/30 uses the stage of its rollout as a performance measure, refers to a roadmap or project plan, and aims for full implementation (Companies and Intellectual Property Commission 2025). Other CIPC materials mention training, procurement, and digitising administration (Companies and Intellectual Property Commission 2025, 2026). This shows real institutional progress, but it is not a fully working examination system.

As CIPC introduces examination, it should ask the ordinary patent questions before using s 25(4)(a). It must test for novelty, inventive step, sufficiency, and claim clarity. It must also consider the exclusions for plant varieties, biological processes, and treatment methods. The morality question only arises when the facts meet the specific wording of the exclusion.

Where a patent file concerns South African genetic resources or associated traditional knowledge, patent administration should record available origin information and identify relevant domestic permit duties. A formal disclosure link will be necessary if South Africa implements the GRATK Treaty. CIPC can improve the visibility of origin information before then.

A published grant is different from a searchable file history. A reader must be able to distinguish an application from a grant, trace its family, and check materials related to its scope and legal status. This review found some records but cannot prove that access is poor across the board. Making that broader claim would need separate institutional research.

Examination, biodiversity disclosure, and clear records are core parts of patent administration, not just minor tasks. They reduce weak grants, link claims to how resources are governed, and help affected people see who has claimed what. None of these tasks requires CIPC to make a general ruling on the morality of cannabis.

## Conclusions

The *Prince* decision should be interpreted narrowly. The Court protected a specific area of adult private conduct on privacy grounds. It did not rule that cannabis is inherently moral. Official sources indicate that the 2024 Act had not yet commenced at the time of writing (Minister of Justice and Constitutional Development and Others v Prince 2018; Cannabis for Private Purposes 2024; Department of Justice and Constitutional Development 2026). Decriminalisation changes the legal background but does not settle the moral debate.

The main patent conclusion still holds after that correction. Even if cannabis is assumed to be morally controversial, s 25(4)(a) does not ban every cannabis-related invention. The question is whether publication or exploitation of the specific invention would generally be expected to encourage offensive or immoral behaviour. The word 'cannabis' alone cannot answer that question.

Refusal is still possible if the legal test is met. The usual grounds for invalidity also still apply. Novelty, inventive step, sufficiency, excluded subject matter, and the difference between plant varieties and technical inventions must all be properly considered. In any case, a patent does not permit conduct that cannabis, medicines, agricultural, or criminal laws ban.

Comparative law is helpful in specific ways. Under the TRIPS Agreement, illegality alone is not enough to trigger the *ordre public* or morality exclusion. Materials from the EPO show why evidence, the specific invention, and lawful ways of using it are important. Zimbabwe provides the closest regional legal comparison. ARIPO, meanwhile, confirms that regional examination and domestic law work together, and do not always need separate national approval. None of these rules can be simply copied.

The problem of fair distribution lies elsewhere. Lesotho and Zimbabwe show that licensed production can grow without wider participation or transparent ownership of related technologies. Malawi's ratification of the GRATK Treaty and South Africa's biodiversity laws instead point towards origin disclosure and benefit sharing. A morality-based objection should not be used to obscure these issues.

South Africa's institutional priorities arise from this difference. CIPC must continue building capacity for substantive search and examination while accurately describing its current progress. It must also make patent records easier to search and understand. Patent administration should link relevant files to biodiversity permits, origin information, and benefit-sharing processes. Better information would help applicants, communities, and policymakers assess technical validity and the distribution of value.

The result is neither a free-for-all on cannabis patents nor a total ban. Decriminalisation based on privacy does not end the moral debate. It does, however, expose the weakness of a categorical exclusion. Section 25(4)(a) must be applied to the specific invention. The laws and institutions designed for those tasks should then address patent quality, regulatory approval, genetic resource disclosure, and inclusive value sharing.

## Data Availability

No datasets were generated or analysed during the current study.

## Abbreviations

ARIPO: African Regional Intellectual Property Organization
CBD: Cannabidiol
CIPC: Companies and Intellectual Property Commission
EPC: European Patent Convention
EPO: European Patent Office
GRATK: Genetic Resources and Associated Traditional Knowledge
PCT: Patent Cooperation Treaty
SSE: Substantive search and examination
THC: Tetrahydrocannabinol
TRIPS: Agreement on Trade-Related Aspects of Intellectual Property Rights
WIPO: World Intellectual Property Organization

## References

[CR1] African Regional Intellectual Property Organization. Harare Protocol on Patents and Industrial Designs (2023 text). https://www.aripo.org/storage/resources-protocols/1675437439_Harare-Protocol-on-Patents-and-Industrial-Designs-2023.pdf. Accessed 10 Apr 2026.

[CR2] Affordable Medicines Trust and Others v Minister of Health and Another [2005] ZACC 3; 2006 (3) SA 247 (CC). Constitutional Court of South Africa. https://collections.concourt.org.za/items/057f6f7b-1ee7-4c41-824a-c4987e2ad390. Accessed 22 Jul 2026.

[CR3] African Regional Intellectual Property Organization. 2024 IP filing trends: key insights from ARIPO's top applicants. 2025. https://www.aripo.org/storage/media/1749626648_ARIPO%20Filings%202024%20Highlights.pdf. Accessed 10 Apr 2026.

[CR4] African Regional Intellectual Property Organization. Harare Protocol on Patents, Utility Models and Industrial Designs (2025 consolidated text). https://www.aripo.org/storage/resources-protocols/1740752895_Harare%20Protocol%20on%20Patents%2C%20Utility%20Models%20and%20Industrial%20Designs%20%282025%29.pdf. Accessed 22 Jul 2026.

[CR5] African Regional Intellectual Property Organization. Patents. https://www.aripo.org/ip-services/patents. Accessed 10 Apr 2026.

[CR6] Agreement on Trade-Related Aspects of Intellectual Property Rights, Marrakesh Agreement Establishing the World Trade Organization, Annex 1C, 1994. https://www.wto.org/english/docs_e/legal_e/27-trips_01_e.htm. Accessed 10 Apr 2026.

[CR7] Amendments to the Regulations on Bioprospecting, Access and Benefit-Sharing, General Notice 447 of 2015, Government Gazette 38809, 19 May 2015. https://www.gov.za/sites/default/files/gcis_document/201505/38809gen447.pdf. Accessed 22 Jul 2026.

[CR8] Barkhuizen v Napier [2007] ZACC 5; 2007 (5) SA 323 (CC). https://www.saflii.org/za/cases/ZACC/2007/5.html. Accessed 10 Apr 2026.

[CR9] Bloomer J. Turning cannabis into cash: agrarian change and Lesotho's evolving experience. EchoGéo. 2019;48. 10.4000/echogeo.17612.

[CR10] Cannabis for Private Purposes Act 7 of 2024. https://www.parliament.gov.za/storage/app/media/Acts/2024/Act_7_of_2024_Cannabis_for_Private_Purposes_Act.pdf. Accessed 10 Apr 2026.

[CR11] Companies and Intellectual Property Commission. Terms of Reference, Bid No. 02–2025/2026–002. 2025. https://www.cipc.co.za/wp-content/uploads/2025/04/Annexure-H-CIPC-Bid-No-02-2025-2026-002.pdf. Accessed 10 Apr 2026.

[CR12] Companies and Intellectual Property Commission. Revised Strategic Plan 2025/26–2029/30. 2026. https://www.cipc.co.za/wp-content/uploads/2026/04/2025-30-CIPC-Revised-Strategic-Plan_vf_20260130.pdf. Accessed 22 Jul 2026.

[CR13] Companies and Intellectual Property Commission. A note from the Patent Office. https://www.cipc.co.za/?p=17839. Accessed 10 Apr 2026.

[CR14] Companies and Intellectual Property Commission. Practice Notice 1 of 2026: Patent stakeholders - electronic issuance of digital patents and patents of addition. 2026. https://www.cipc.co.za/wp-content/uploads/2026/01/Practice-Notice-1-of-2026-combined.pdf. Accessed 10 Apr 2026.

[CR15] Constitution of the Republic of South Africa, 1996. https://www.gov.za/documents/constitution-republic-south-africa-1996. Accessed 17 May 2026.

[CR16] Convention on the Grant of European Patents, Article 53. European Patent Office. https://www.epo.org/en/legal/epc/2020/a53.html. Accessed 10 Apr 2026.

[CR17] Department of Justice and Constitutional Development. Invitation for public comments on Draft Cannabis Regulations. Government Gazette 54061, R 7067, 2 February 2026. https://www.justice.gov.za/legislation/invitations/20260202-InviteToComment-Draft%20Cannabis%20Regulations.pdf. Accessed 22 Jul 2026.

[CR18] Dube I, Makola ZS, Ntoyanto N, Msiza C. Investigating small-scale farmers and barriers to entry in cannabis cultivation in South Africa. Politeia. 2023;42(1):1–21. 10.25159/2663-6689/15002.

[CR19] Enever v Barloworld Equipment South Africa, A Division of Barloworld South Africa (Pty) Ltd (JA86/22) [2024] ZALAC 12; [2024] 6 BLLR 562 (LAC); (2024) 45 ILJ 1554 (LAC). https://www.saflii.org/za/cases/ZALAC/2024/12.html. Accessed 10 Apr 2026.

[CR20] EP1536810B1. Extraction of pharmaceutically active cannabinoids from plant materials. Google Patents. https://patents.google.com/patent/EP1536810B1/en. Accessed 22 Jul 2026.

[CR21] EP2161262A1. THCV and THCV extracts derived from plant material. Google Patents. https://patents.google.com/patent/EP2161262A1/en. Accessed 22 Jul 2026.

[CR22] European Patent Office. National law relating to the EPC. 23rd ed. 2024. https://www.epo.org/en/legal/national-law. Accessed 10 Apr 2026.

[CR23] European Patent Office. National validation in the extension/validation states. https://www.epo.org/en/applying/european/validation. Accessed 10 Apr 2026.

[CR24] European Patent Office. Guidelines for Examination, G-II, 4.1 Matter contrary to ordre public or morality. 2026. https://www.epo.org/en/legal/guidelines-epc/2026/g_ii_4_1.html. Accessed 22 Jul 2026.

[CR25] European Union Drugs Agency. European drug report 2024: cannabis. Lisbon: EUDA; 2024.

[CR26] G 0001/98 (Transgenic plant/NOVARTIS II) of 20.12.1999. European Patent Office. https://www.epo.org/en/boards-of-appeal/decisions/g980001ex1. Accessed 10 Apr 2026.

[CR27] Ghana. Patent Act 2003 (Act 657), s 3(2)(d). WIPO Lex. https://www.wipo.int/wipolex/en/legislation/details/9426. Accessed 22 Jul 2026.

[CR28] Google. Google Patents database. https://patents.google.com/. Accessed 22 Jul 2026.

[CR29] Government of South Africa. MEC Ivan Meyer on jobs in the cannabis and hemp industry. 2025. https://www.gov.za/news/media-statements/mec-ivan-meyer-jobs-cannabis-and-hemp-industry-20-mar-2025. Accessed 10 Apr 2026.

[CR30] Grand View Research. Legal Marijuana Market Size To Reach $102.2Bn By 2030. 2024. https://www.grandviewresearch.com/press-release/global-legal-marijuana-market. Accessed 17 May 2026.

[CR31] Laugh It Off Promotions CC v South African Breweries International (Finance) BV t/a Sabmark International and Another [2005] ZACC 7; 2006 (1) SA 144 (CC). Constitutional Court of South Africa. https://collections.concourt.org.za/items/90953b1a-a382-47cd-a048-0a9eb073478f. Accessed 22 Jul 2026.

[CR32] Lesotho. Industrial Property Order 1989 (Order No. 5 of 1989, as amended by Act No. 4 of 1997), s 5(8). WIPO Lex. https://www.wipo.int/wipolex/en/legislation/details/5874. Accessed 22 Jul 2026.

[CR33] Lesotho National Development Corporation. Medicinal Cannabis. 2025. https://lndc.org.ls/medicinal-cannabis/. Accessed 10 Apr 2026.

[CR34] Lubaale EC, Mavundla SD. Decriminalisation of cannabis for personal use in South Africa. Afr Hum Rights Law J. 2019;19(2):819–42. 10.17159/1996-2096/2019/v19n2a13.

[CR35] Malawi. Cannabis Regulation Act 2020 (Act No. 6 of 2020). Malawi Legal Information Institute. https://malawilii.org/akn/mw/act/2020/6/eng@2020-05-08. Accessed 22 Jul 2026.

[CR36] Malawi. Patents Act [Chapter 49:02], s 18. WIPO Lex. https://www.wipo.int/wipolex/en/legislation/details/9337. Accessed 22 Jul 2026.

[CR37] Mbiada CJT, Joubert G, Ndlovu L. When cannabis use privately finds itself in the workplace: a reflection on Enever v Barloworld Equipment, a division of Barloworld SA (Pty) Ltd (2024) 45 ILJ 1554 (LAC). Int J Res Bus Soc Sci. 2024;13(9):299–304. 10.20525/ijrbs.v13i9.3812.

[CR38] Medicines Control Authority of Zimbabwe. Medicinal Cannabis. https://www.mcaz.co.zw/medicinal-cannabis/. Accessed 10 Apr 2026.

[CR39] Minister of Justice and Constitutional Development and Others v Prince; National Director of Public Prosecutions and Others v Rubin; National Director of Public Prosecutions and Others v Acton and Others [2018] ZACC 30; 2018 (6) SA 393 (CC). Constitutional Court of South Africa. https://collections.concourt.org.za/bitstreams/37f05063-5402-45ef-bed8-ceb66730ed3d/download. Accessed 21 May 2026.

[CR40] Narcotics Control Commission (Ghana). Cannabis Regulations. 2026. https://www.ncc.gov.gh/cannabis-regulations/. Accessed 10 Apr 2026.

[CR41] National Environmental Management: Biodiversity Act 10 of 2004, as amended. https://www.gov.za/documents/national-environmental-management-biodiversity-act-0. Accessed 22 Jul 2026.

[CR42] Oriakhogba DO, Okorie CI, Lavhengwa LS. South African dagga: piping on the related intellectual property rights and indigenous knowledge systems. Int J Intellect Prop Manag. 2024;14(5):459–84. 10.1504/IJIPM.2024.141543.

[CR43] Patents Act 57 of 1978, as amended. https://www.thedtic.gov.za/wp-content/uploads/patent_act.pdf. Accessed 22 Jul 2026.

[CR44] Rusenga C, Klantschnig G, Carrier N, Howell S. Business as usual? Cannabis legalisation and agrarian change in Zimbabwe. J Peasant Stud. 2024;51(4):982–1001. 10.1080/03066150.2024.2326568.

[CR45] T 0315/03 (Transgenic animals/HARVARD) of 06.07.2004. European Patent Office. https://www.epo.org/en/boards-of-appeal/decisions/t030315ex1. Accessed 10 Apr 2026.

[CR46] Thetsane RM. Envisaging challenges for the emerging medicinal Cannabis sector in Lesotho. J Cannabis Res. 2024;6:23. 10.1186/s42238-024-00229-9. PMC11097424 38755701

[CR47] United Nations Office on Drugs and Crime. World drug report 2025: key findings. Vienna: UNODC; 2025.

[CR48] Western Cape Department of Agriculture. Western Cape unveils Cannabis Implementation Plan for Agriculture. 2023. https://www.elsenburg.com/western-cape-unveils-cannabis-implementation-plan-for-agriculture/. Accessed 10 Apr 2026.

[CR49] WO2024227244A1. Genetic markers for powdery mildew resistance in cannabis and related methods. Google Patents. https://patents.google.com/patent/WO2024227244A1/en. Accessed 22 Jul 2026.

[CR50] World Intellectual Property Organization. Malawi is First to Ratify WIPO Treaty on IP, Genetic Resources and Associated Traditional Knowledge. 2024. https://www.wipo.int/pressroom/en/articles/2024/article_0019.html. Accessed 10 Apr 2026.

[CR51] World Intellectual Property Organization. WIPO Treaty on Intellectual Property, Genetic Resources and Associated Traditional Knowledge. WIPO Lex; 2024. https://www.wipo.int/wipolex/en/text/593055. Accessed 10 Apr 2026.

[CR52] World Intellectual Property Organization. WIPO Treaty on Intellectual Property, Genetic Resources and Associated Traditional Knowledge. 2024. https://www.wipo.int/en/web/treaties/ip/gratk/index. Accessed 10 Apr 2026.

[CR53] World Intellectual Property Organization. G 1/98, European Patent Organisation. WIPO Lex. https://www.wipo.int/wipolex/en/judgments/details/2624. Accessed 10 Apr 2026.

[CR54] World Intellectual Property Organization. PATENTSCOPE database. https://www.wipo.int/en/web/patentscope. Accessed 22 Jul 2026.

[CR55] World Intellectual Property Organization. WIPO Treaty on Intellectual Property, Genetic Resources and Associated Traditional Knowledge: status on 13 March 2026. https://www.wipo.int/documents/d/treaties/docs-en-gratk.pdf. Accessed 22 Jul 2026.

[CR56] Wyse J, Luria G. Trends in intellectual property rights protection for medical cannabis and related products. J Cannabis Res. 2021;3:1. 10.1186/s42238-020-00057-7. PMC7819315 33526141

[CR57] ZA200802767B. Process for production of delta-9-tetrahydrocannabinol. Google Patents. https://patents.google.com/patent/ZA200802767B/en. Accessed 22 Jul 2026.

[CR58] ZA201906516B. Process for purification and separation of cannabinoids from dried hemp and cannabis leaves. Google Patents. https://patents.google.com/patent/ZA201906516B/en. Accessed 22 Jul 2026.

[CR59] ZA202205648B. Cannabinoid nanomicelle preparation and method for preparing same. Google Patents. https://patents.google.com/patent/ZA202205648B/en. Accessed 22 Jul 2026.

[CR60] Zimbabwe. Patents Act [Chapter 26:03], s 13. WIPO Lex. https://www.wipo.int/wipolex/en/legislation/details/8942. Accessed 22 Jul 2026.

